# Ethnicity and outcomes for patients with gastrointestinal disorders attending an emergency department serving a multi-ethnic population

**DOI:** 10.1186/s12916-024-03490-0

**Published:** 2024-07-02

**Authors:** Christopher A. Martin, Tim Coats, Manish Pareek, Kamlesh Khunti, Ruw Abeyratne, Nigel J. Brunskill

**Affiliations:** 1 Department of Cardiovascular Sciences, University of Leicester, Leicester, UK; 2 Department of Nephrology, University Hospitals of Leicester NHS Trust, Leicester, UK; 3 Department of Respiratory Sciences, University of Leicester, Leicester, UK; 4 Department of Infection and HIV Medicine, University Hospitals of Leicester NHS Trust, Leicester, UK; 5 Development Centre for Population Health, University of Leicester, Leicester, UK; 6 Department of Emergency and Specialist Medicine, University Hospitals of Leicester NHS Trust, Leicester, UK; 7 Diabetes Research Centre, Centre for Ethnic Health Research, University of Leicester, Leicester, UK; 8 Department of Corporate Medicine, University Hospitals of Leicester NHS Trust, Leicester, UK

**Keywords:** Ethnicity, Gastrointestinal, Admission, Investigation, Emergency care

## Abstract

**Background:**

Ethnic inequalities in acute health acute care are not well researched. We examined how attendee ethnicity influenced outcomes of emergency care in unselected patients presenting with a gastrointestinal (GI) disorder.

**Methods:**

A descriptive, retrospective cohort analysis of anonymised patient level data for University Hospitals of Leicester emergency department attendees, from 1 January 2018 to 31 December 2021, receiving a diagnosis of a GI disorder was performed. The primary exposure of interest was self-reported ethnicity, and the two outcomes studied were admission to hospital and whether patients underwent clinical investigations. Confounding variables including sex and age, deprivation index and illness acuity were adjusted for in the analysis. Chi-squared and Kruskal–Wallis tests were used to examine ethnic differences across outcome measures and covariates. Multivariable logistic regression was used to examine associations between ethnicity and outcome measures.

**Results:**

Of 34,337 individuals, median age 43 years, identified as attending the ED with a GI disorder, 68.6% were White. Minority ethnic patients were significantly younger than White patients. Multiple emergency department attendance rates were similar for all ethnicities (overall 18.3%). White patients had the highest median number of investigations (6, IQR 3–7), whereas those from mixed ethnic groups had the lowest (2, IQR 0–6). After adjustment for age, sex, year of attendance, index of multiple deprivation and illness acuity, all ethnic minority groups remained significantly less likely to be investigated for their presenting illness compared to White patients (Asian: aOR 0.80, 95% CI 0.74–0.87; Black: 0.67, 95% CI 0.58–0.79; mixed: 0.71, 95% CI 0.59–0.86; other: 0.79, 95% CI 0.67–0.93; *p* < 0.0001 for all). Similarly, after adjustment, minority ethnic attendees were also significantly less likely to be admitted to hospital (Asian: aOR 0.63, 95% CI 0.60–0.67; Black: 0.60, 95% CI 0.54–0.68; mixed: 0.60, 95% CI 0.51–0.71; other: 0.61, 95% CI 0.54–0.69; *p* < 0.0001 for all).

**Conclusions:**

Significant differences in usage patterns and disparities in acute care outcomes for patients of different ethnicities with GI disorders were observed in this study. These differences persisted after adjustment both for confounders and for measures of deprivation and illness acuity and indicate that minority ethnic individuals are less likely to be investigated or admitted to hospital than White patients.

**Supplementary Information:**

The online version contains supplementary material available at 10.1186/s12916-024-03490-0.

## Background

Identifying and driving down health inequalities is a key goal as set out in the NHS Long Term Plan [1]. Ethnic inequalities in health are well described in the UK, where individuals from minority ethnic backgrounds generally experience worse health outcomes than White British people [2]. This is particularly true for long term conditions such as type 2 diabetes and cardiovascular diseases which disproportionately affect South Asian individuals [3–7]. The evidence for ethnicity-related inequalities in acute care is less well researched, but studies of emergency hospital admissions indicate a complex relationship between ethnicity and the risk of admission where non-White patients tend to be younger and experience a lower mortality risk than White patients [8], but with ethnic minorities being at higher risk of admission for some illnesses [9].

The underlying causes of these ethnic differences in health outcomes are complex and poorly understood with a variety of potentially contributing and intersecting factors such as multiple long-term conditions and social determinants of health. It is important for providers of acute care to identify and investigate differences in the processes of care and their outcomes for all users of their services to enable the effective planning of care pathways and the appropriate targeting of healthcare interventions to all sectors of the community.

Gastrointestinal (GI) disorders represent the second most common classifiable diagnoses in patients presenting to English hospital Accident and Emergency Departments (ED) (fractures/dislocations/joint injuries being the most common) [10], and their assessment and treatment is associated with high healthcare costs.

Therefore, as part of a Trust-wide programme of clinical service inequality analyses, we examined how attendee ethnicity influenced outcomes related to the processes of acute emergency care, focusing on unselected patients of various ethnicities with presenting to the ED at a busy acute Trust in England with a GI disorder.

## Methods

University Hospitals of Leicester NHS Trust (UHL) provides acute care services to a population of over 1.2 million across Leicester, Leicestershire and Rutland [11]. This population is ethnically diverse such that within the City of Leicester, more than 50% of residents have a minority ethnic background, with the highest number being of South Asian heritage [12]. In contrast, the surrounding County of Leicestershire population is of 87.5% White ethnicity [13]. The Accident and Emergency Department (ED) at UHL had around 242,000 attendances in the 12 months from the beginning of January to the end of December 2019.

### Study design and data source

In this descriptive, retrospective cohort study, anonymised patient level data were extracted from the hospital’s electronic data systems. A fully anonymised dataset was then provided to the investigators. This analysis of anonymised, routine patient data was approved by the UHL Data Protection Officer/Head of Privacy and registered as a service evaluation by the UHL Clinical Audit and Effectiveness Team (reference number 11675). We conducted and reported this service evaluation following the RECORD (REporting of studies Conducted using Observational Routinely Collected Data (RECORD) checklist (Additional file 1: Table S1) [14].

### Study population

All patients who attended the UHL ED and received a coded diagnosis for a gastrointestinal or hepatobiliary illness (hereafter referred to as a ‘GI disorder’, see Additional file 1; Table S2 for diagnostic codes included) in the electronic patient record (EPR) between 1 January 2018 and 31 Dec 2021 were identified and formed the cohort population. Specified patient level data for this cohort (see below) were then extracted from the UHL data warehouse. For patients with multiple attendances for with a GI disorder in this period, the number of attendances was recorded, but only the first attendance was studied. Sociodemographic factors (age and sex) were extracted along with the day and time of ED attendance and the length of time in the ED. The date of attendance at the ED was collapsed into year of attendance (2018 to 2021).

### Exposure, outcomes and covariables

The primary exposure of interest was patient self-reported ethnicity. Codes for self-reported ethnicity recorded in the EPR (Additional file 1; Table S3) were used to derive a five-level ethnicity variable using the same broad ethnic groupings as the UK Office for National Statistics: White, Black, Asian, mixed, or other [15]. Initial dynamic priority score (DPS) and initial early warning scores were extracted as measures of acuity of illness presentation.

Two outcomes were evaluated for their association with ethnicity, firstly whether the patient was admitted to hospital after presentation to the ED (binary variable: admitted [1] vs not admitted [0]) and secondly whether the patient underwent any clinical investigations in the ED (binary variable: underwent any investigation [1] vs did not undergo any investigations [0]). For a list of investigations and procedures, see Additional file 1: Table S3.

Data describing variables which could potentially confound the relationship between ethnicity and decisions relating to hospital admission or investigation were also collected. These included sex and age at presentation (categorised into 0–17 years, 18–29 years, 30–59 years, ≥ 60 years). We collected data relating to breaches of the national 4-h emergency department waiting time target [16]. Deprivation in residential areas was determined using the index of multiple deprivation (IMD) derived from the patient postcode. The IMD is the official measure of relative deprivation for England [17] where small residential areas are ranked based on 7 domains (income, employment, education, health, crime, barriers to housing/services and living environment). Ranks were collapsed into quintiles. Severity of illness on presentation as measured by the National Early Warning Score [18] or Paediatric Early Warning Score [19] (hereafter referred to as EWS) on presentation to the ED. The EWS is an aggregate scoring system used to classify the severity of an acute illness based on routinely gathered physiological parameters. We also collected the DPS assigned to every ED attendee as a local prioritisation system [20].

We produced descriptive statistics for some additional common NHS metrics including number of four-hour breaches and ED waiting times to fully describe the underlying data. These metrics were not used as outcome measures in the adjusted analyses to avoid overlap between factors associated with total time in ED and decisions to admit.

### Statistics

Categorical variables were summarised as frequency and percentage and continuous variables as median and interquartile range (IQR). Chi-squared tests and Kruskal–Wallis tests were used to examine ethnic differences across outcome measures and covariates for categorical and continuous variables respectively. The number of ED attendances were plotted against the number and proportion of patients admitted over the study period.

Multivariable logistic regression was used to examine the association between ethnicity and our binary outcome measures and present results as adjusted odds ratios (aORs) and 95% confidence intervals (95% CIs). Three models were constructed for each outcome: model 1 was adjusted for age, sex and year of attendance; model 2 included all variables in model 1 plus EWS; model 3 included all variables in model 2 plus IMD quintile. This sequential adjustment was undertaken to examine whether ethnic differences in severity at presentation and/or deprivation might explain any age/sex adjusted associations between ethnicity and our outcome measures.

The frequency and proportion of observations with missing data was calculated in each of the variables used in the analysis. Multiple imputation by chained equations to impute missing covariate data was used in all logistic regression models. Imputation models contained all variables used in the analysis except the one being imputed, including the outcome measures. Rubin’s Rules were used to combine parameter estimates and standard errors from 10 imputations into a single set of results [21]. To investigate the impact of using multiple imputation on our results, a sensitivity analysis was undertaken using listwise deletion, excluding those with missing covariate data in any variable used in the models.

All analyses were conducted using Stata 17 (StataCorp. 2017. Stata Statistical Software: Release 17.0 College Station, TX: StataCorp LLC).

## Results

### Cohort characteristics

**Fig. 1:**
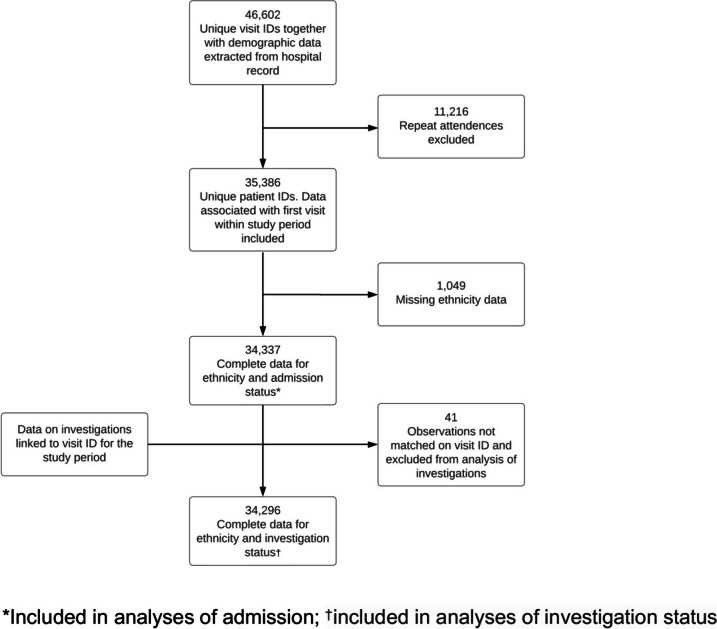
Formation of the analysed cohort. ‘*’ symbol indicates the following: included in analyses of admission; ‘†’symbol indicates the following: included in analyses of investigation status

In total, there were 46,602 visits to the ED with a GI disorder in the study period. After exclusion of repeat attendances, 34,337 individuals were identified as attending the ED with a GI disorder with fully coded ethnicity and admission data, of whom 34,296 had complete data for ethnicity and investigations (Fig. 1). Baseline characteristics and demographics of the cohort are shown in Table 1. Ethnicity coding was complete for most attendees in the study period with 1049 (2.9%) attendees having missing or ‘not stated’ ethnicity data. Of those with coded ethnicity, 23,548 (68.6%) were White, 7450 (21.7%) were Asian, 1410 (4.1%) were Black, 722 (2.1%) were of mixed ethnicity and 1207 (3.5%) reported other ethnicities.

**Table 1.** Description of the cohort stratified by ethnic group

Variable	Total* N = 34,337	White *N* = 23,548 (68.6)	Asian *N* = 7450 (21.7)	Black *N* = 1410 (4.1)	Mixed *N* = 722 (2.1)	Other *N* = 1207 (3.5)	*p* value^†^
**Age**, med (IQR)	43 (23–67)	49 (26–72)	37 (20–57)	30 (18–45)	19 (7–34)	29 (17–44)	< 0.001
**Age**							
0 to 17 years	6066 (17.7)	3375 (14.3)	1696 (22.8)	335 (23.8)	338 (46.8)	322 (26.7)	< 0.001
18 to 29 years	5693 (16.6)	3649 (15.5)	1209 (16.2)	366 (26.0)	165 (22.9)	304 (25.2)	
30 to 59 years	11,621 (33.8)	7562 (32.1)	2854 (38.3)	558 (39.6)	195 (27.0)	452 (37.5)	
≥ 60 years	10,955 (31.9)	8961 (38.1)	1691 (22.7)	151 (10.7)	24 (3.3)	128 (10.6)	
Missing	2 (0.0)	1(0.0)	0 (0.0)	0 (0.0)	0 (0.0)	1 (0.1)	
**Sex**, ***N*** **(%)**							
Female	19,207 (55.9)	13,298 (56.5)	4087 (54.9)	856 (60.7)	405 (56.1)	561 (46.5)	< 0.001
Male	15,130 (44.1)	10,250 (43.4)	3363 (45.1)	554 (39.3)	317 (43.9)	646 (53.5)	
**IMD quintile, *N*** **(%)**							
1 (most deprived)	6740 (19.6)	4026 (17.1)	1581 (21.2)	575 (40.8)	227 (31.4)	331 (27.4)	< 0.001
2	7076 (20.6)	3911 (16.6)	2378 (31.9)	321 (22.8)	139 (19.3)	327 (27.1)	
3	5573 (16.2)	3929 (16.7)	1239 (16.6)	145 (10.3)	107 (14.8)	153 (12.7)	
4	6540 (19.1)	5337 (22.7)	900 (12.1)	95 (6.7)	105 (14.5)	103 (8.5)	
5 (least deprived)	5914 (17.2)	5062 (21.5)	630 (8.5)	64 (4.5)	76 (10.5)	82 (6.8)	
Missing	2494 (7.3)	1296 (5.5)	722 (9.7)	210 (14.9)	68 (9.4)	211 (17.5)	
**Number (%) with repeated visits in study period**	6268 (18.3)	4357 (18.5)	1330 (17.9)	255 (18.1)	136 (18.8)	190 (15.7)	0.13
**Total time in ED** (minutes), med (IQR)	269 (181–412)	284 (191–427)	240 (168–391)	229 (150–347)	203 (134–301)	234 (160–361)	< 0.001
**Number (%) of 4 h breaches**	18,668 (54.4)	13,526 (57.4)	3705 (49.7)	622 (44.1)	253 (35.0)	562 (46.4)	< 0.001
**Initial EWS, *N*** **(%)**							
0	14,956 (43.6)	9941 (42.2)	3394 (45.6)	695 (49.3)	355 (49.2)	571 (47.4)	< 0.001
1 to 4	14,620 (42.6)	10,193 (43.3)	3119 (41.9)	525 (37.2)	299 (41.4)	484 (40.1)	
5 to 6	777 (2.3)	599 (2.5)	132 (1.8)	13 (0.9)	11 (1.5)	22 (1.8)	
≥ 7	461 (1.3)	395 (1.7)	51 (0.7)	8 (0.6)	0 (0.0)	7 (0.6)	
Missing	3523 (10.3)	2420 (10.3)	754 (10.1)	169 (12.0)	57 (7.8)	123 (10.2)	
**Initial DPS, *N*** **(%)**							
1 (highest priority)	200 (0.6)	163 (0.7)	24 (0.3)	4 (0.3)	2 (0.4)	7 (0.6)	< 0.001
2	3453 (10.1)	2605 (11.1)	563 (7.6)	109 (7.7)	77 (10.7)	99 (8.2)	
3	30,572 (89.0)	20,732 (88.0)	6821 (91.6)	1283 (91.0)	639 (88.5)	1097 (90.9)	
4 (lowest priority)	104 (0.3)	41 (0.2)	42 (0.6)	13 (0.9)	4 (0.6)	4 (0.3)	
Missing	8 (0.0)	7 (0.0)	0 (0.0)	1 (0.1)	0 (0.0)	0 (0.0)	
**Investigations,*N*** **(%)**							
No investigations	4409 (12.8)	2445 (10.4)	1234 (16.6)	289 (20.5)	204 (28.3)	237 (19.6)	< 0.001
Had investigations	29,887 (87.0)	21,075 (89.5)	6208 (83.3)	1117 (79.2)	517 (71.6)	970 (80.4)	
Missing	41 (0.12)	28 (0.1)	8 (0.1)	4 (0.3)	1 (0.1)	0 (0.0)	
**Number of investigations,** med (IQR)	5 (3–7)	6 (3–7)	5 (1–7)	4 (1–6)	2 (0–6)	4 (1–7)	< 0.001
**Admission, *N*** **(%)**							
Not admitted	15,365 (44.8)	9257 (39.3)	4085 (54.8)	843 (59.8)	463 (64.1)	717 (59.4)	< 0.001
Admitted	18,972 (55.3)	14,291 (60.7)	3365 (45.2)	567 (40.2)	259 (35.9)	490 (40.6)	

*DPS* Dynamic priority score, *IMD* Index of multiple deprivation, *IQR* Interquartile range, *EWS* Early warning score

^*^Describes only those with complete ethnicity data. Those with missing ethnicity data and those coded as ‘not stated’ are excluded (*N* = 1049)

^†^Comparison between ethnic groups was by chi-squared test for categorical variables and by Kruskal–Wallis test for continuous variables

The median age of attendees was 43 years (IQR 23–67 years), but patients from minority ethnic groups were significantly younger (*p* < 0.001,Table 1) than White attendees (Asian: 37 years [20–57 years], Black 30 years [18–45 years], mixed: 19 years [7–34 years], other: 29 years [17–44 years, White: 49 years [26–72 years]). Females comprised 55.7% of all attendees with a similar pattern across all ethnic groups. Minority ethnic attendees were significantly more likely to be from the most deprived areas (*p* < 0.001, Table 1), whereas White attendees were more equally distributed across all groups of deprivation.

The median time spent in ED for all attendees was 269 min [IQR 181–412 min], and people of White ethnicity had the longest time spent in ED (Asian: 240 min [168–391 min], Black: 229 min [150–347 min], mixed: 203 min [134–301 min], other: 234 min [160–361 min], White: 284 min [191–427 min]) (Table 1). The highest proportion of 4-h waiting target breaches occurred in the White group (White 57.4%, Asian 49.7%, Black 44.1%, mixed 35.0% and other 46.4%). In terms of acuity of presentation, White patients presented with significantly higher illness acuity than ethnic minority patients, as measured by both EWS and DPS (*p* < 0.001, Table 1).

### ED attendance patterns across the study period

**Fig. 2:**
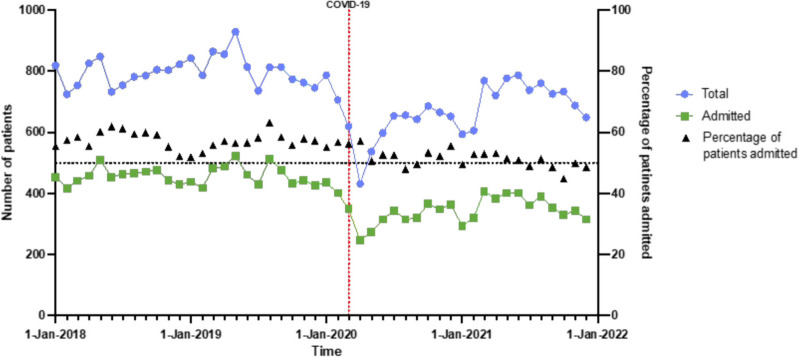
Monthly attendances and admissions for patients with GI disorders over the study period

The pattern of ED attendances by patients with GI disorders is shown in Fig. 2. There was a steep decline in the overall numbers of both attendances and admissions before the first national UK lockdown in March 2020. These numbers were still lower at the end of 2021 compared to pre-pandemic levels. However, the proportion of patients admitted showed little change.

Multiple attendances were recorded for 18.3% of all attendees (Table 1). However, there were no significant differences in the rate of multiple attendances between the different ethnic groups.

### Factors influencing performance of clinical investigations

Overall, 29,887 (87.0%) attendees with an GI disorder underwent some form of clinical investigation into their presenting illness (see Additional file 1: Table S3 for investigations studied).

**Fig. 3:**
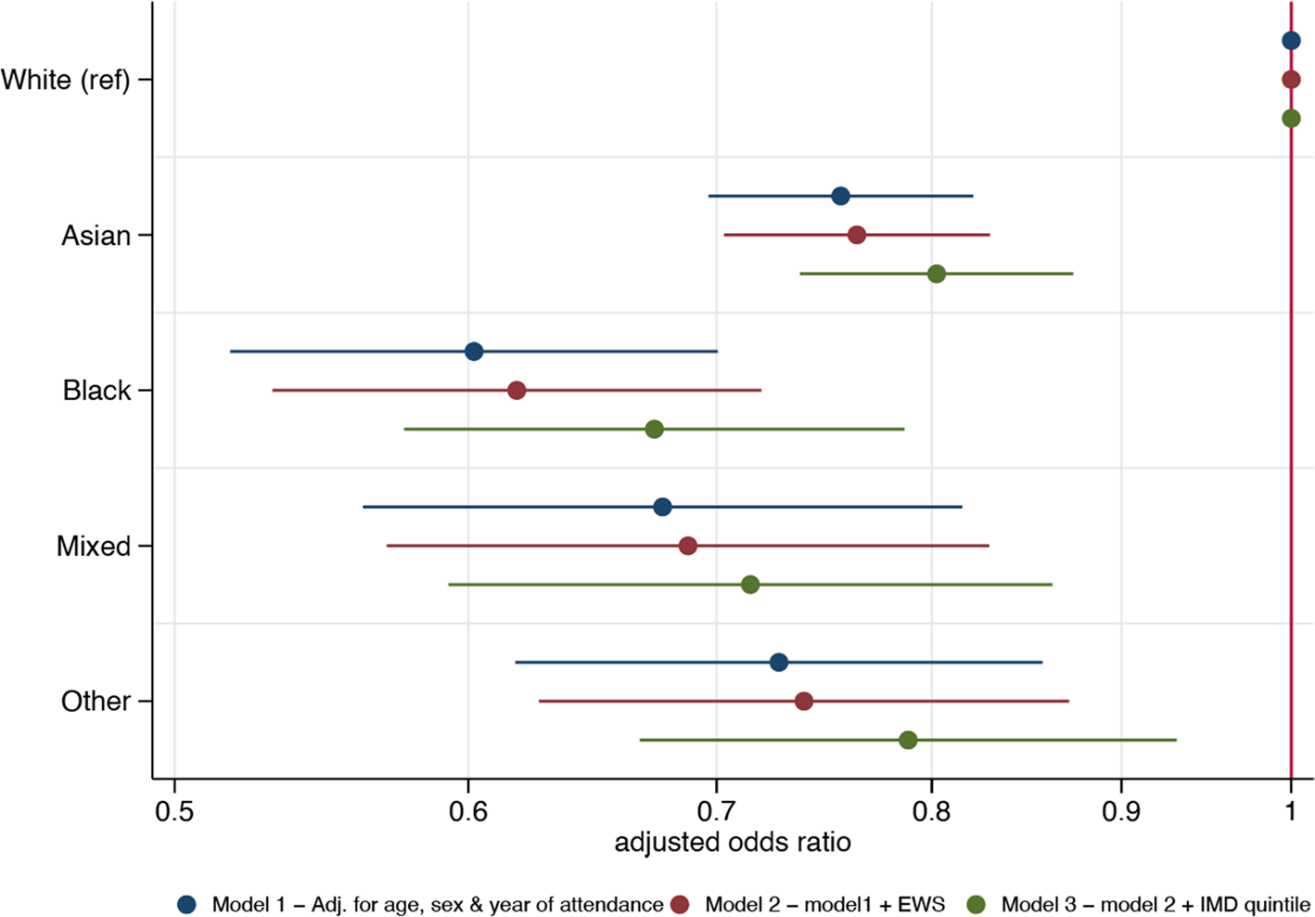
Logistic regression models showing the relationship between ethnicity with having investigations performed following attendance at the emergency department with GI disorders. Figure 3 shows the adjusted odds ratios and 95% confidence intervals for the relationship between ethnicity with undergoing any investigations after presentation to the emergency department with abdominal pain (*n* = 34,296) using the imputed dataset. Three models were constructed; the first (blue) is adjusted for age, sex and the year of first attendance at the ED with abdominal pain during the study period. The second (red) is additionally adjusted for EWS. The third (green) is additionally adjusted for the IMD quintile. Estimates are represented by dots and the 95% confidence interval for the estimate by bars. EWS, early warning score; IMD, index of multiple deprivation; Ref, reference level

A higher proportion of those from White ethnic groups were investigated for their GI disorder than any other ethnic group studied (Table 1). Overall, the median number of investigations per individual attendee was 5 (IQR 3–7). White patients had the highest median investigation count, and those from mixed ethnic groups had the lowest (White: 6 [3,–7], Asian 5 [1,–7], Black: 4 [1,–6], mixed 2 [0–6], other: 4 [1,–7]) (Table 1). Using White attendees as the reference, and after adjustment for year of attendance, age, sex, initial EWS score and IMD quintile, all ethnic minority groups remained significantly less likely to be investigated for their presenting illness (in the fully adjusted model, Asian: aOR 0.80, 95% CI 0.74–0.87; Black: 0.67, 95% CI 0.58–0.79; mixed: 0.71, 95% CI 0.59–0.86; other: 0.79, 95% CI 0.67–0.93; *p* < 0.0001 for all) (Fig. 3 and Additional file 1: Table S4). Children below the age of 18 years of age were significantly less likely to be investigated than older patients (aOR 0.18, 95% CI 0.16–0.20 vs patients aged 18 to 29 years, *p* < 0.0001), and males were less likely to be investigated than females (aOR 0.70, 95% CI 0.65–0.75, *p* < 0.0001) as most females with abdominal symptoms will have a pregnancy test (Additional file 1: Figure S1 and Table S4). There was a general trend such that the aOR for receiving investigations was inversely related to level of deprivation. The least deprived (those from IMD quintile 5) were significantly more likely and the most deprived (IMD quintile 1) were significantly less likely to have investigations than those from IMD quintile 3 (IMD quintile 5: 1.16, 95% CI 1.01–1.32, *p* = 0.03; IMD quintile 1: 0.88, 95% CI 0.78–0.99, *p* = 0.03) (Additional file 1: Figure S1 and Table S4).

### Factors influencing discharges and admissions to hospital

Of the 34,337 patients attending the ED with GI disorders 18,972 (55.3%) were admitted to hospital. Amongst those admitted, median (IQR) ages for each ethnic group were as follows: White: 57 years (32–76 years); Asian: 44 years (26–65 years); Black: 36 years (22–52 years); mixed: 25 years (14–40 years); other: 34 years (21–51 years).

The numbers of such attendees declined sharply in early 2020 corresponding to the start of the COVID-19 pandemic (Fig. 2). There was an accompanying reduction in the number of individuals with GI disorders admitted to the hospital from the ED over this period. Attendees were significantly less likely to be admitted in 2020 and 2022 compared to 2018 and 2019 (Additional file 1: Figure S2 and Table S5).

**Fig. 4:**
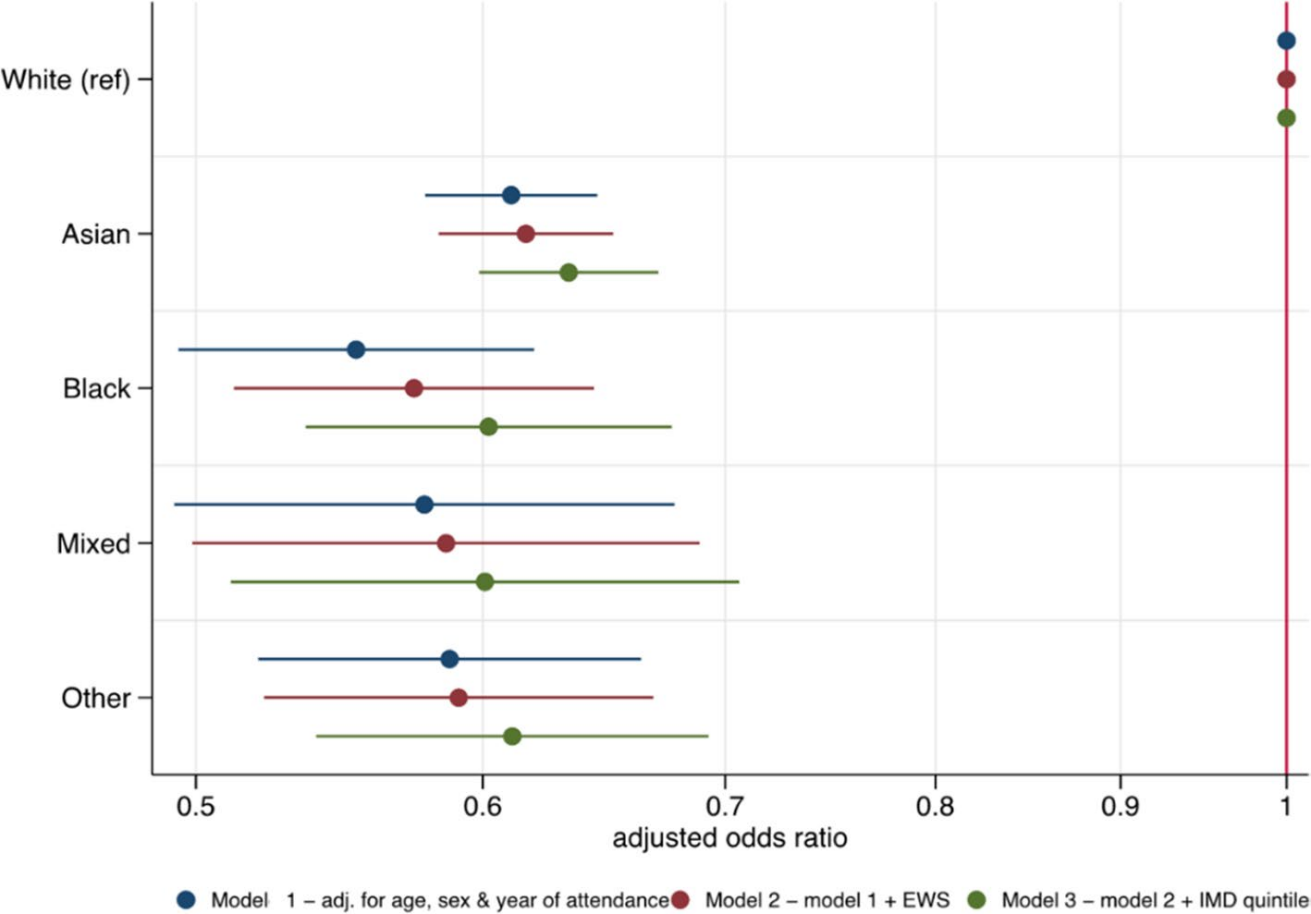
Logistic regression models showing the association between ethnicity and admission following attendance at the emergency department with GI disorders. Figure 4 shows the adjusted odds ratios and 95% confidence intervals for the relationship between ethnicity and admission to hospital after presentation to the emergency department with abdominal pain (*n* = 34,337) using the imputed dataset. Three models were constructed; the first (blue) is adjusted for age, sex and the year of first attendance at the ED with abdominal pain during the study period. The second (red) is additionally adjusted for EWS. The third (green) is additionally adjusted for the IMD quintile. Estimates are represented by dots and the 95% confidence interval for the estimate by bars. *x* axis is on a log scale. EWS, early warning score; IMD, index of multiple deprivation; Ref, reference level

Minority ethnic patients presenting with GI disorders tended to have lower initial EWS and a lower priority DPS than White attendees and were significantly less likely to be admitted to hospital than White patients (*p* < 0.001, Table 1). However, when analyses were adjusted for the variables—age, sex, year of attendance, EWS and IMD quintile—minority ethnic attendees were significantly less likely than those from White ethnic groups to be admitted to hospital in all models tested (in the fully adjusted model, Asian: aOR 0.63, 95% CI 0.60–0.67; Black: 0.60, 95% CI 0.54–0.68; mixed: 0.60, 95% CI 0.51–0.71; other: 0.61, 95% CI 0.54–0.69; *p* < 0.0001 for all) (Fig. 4 and Additional file 1: Table S5). Using patients aged 18–29 years as a reference group, children were significantly less likely to be admitted and older patients significantly more likely to be admitted to hospital (0 to 17 years: aOR 0.59, 95% CI 0.55–0.64, ≥ 60 years: 2.53, 95% CI 2.36–2.71; *p* < 0.0001 for both) (Additional file 1: Figure S2). There was no significant difference in the odds of admission between males and females after adjustment (Additional file 1: Figure S2 and Table S5). Those living in the most deprived IMD quintile were significantly less likely to be admitted to hospital than those in quintile 3 (aOR 0.90, 95% CI 0.83–0.97; *p* = 0.005) (Additional file 1: Figure S2 and Table S5).

In a sensitivity analysis using observations with complete case data, our findings were largely unchanged (Additional file 1: Figures S3 and S4).

## Discussion

Considerable evidence describes health inequalities related to ethnicity [3–7, 22] and several studies demonstrate racial and ethnic disparities in emergency medicine [23–26]; however in the UK, there has been little focus on inequalities in acute hospital care. To our knowledge, this is the largest analysis of outcomes for ED attendees with GI disorders broken down by ethnic group. The chosen outcome measures, directly related to the process of care in the ED, were the frequency of patients with such disorders receiving the clinical investigations common in this clinical setting or the likelihood of being admitted to hospital after ED assessment. We looked at patient-level data from attendances at a busy ED in England including the 2 years before and the nearly 2 years after the commencement of the COVID-19 pandemic and found significant differences in the patterns of usage and disparities in care and outcomes for patients of different ethnicities with GI disorders. Statistical adjustment for potentially confounders, including socioeconomic factors and differences in illness acuity, attenuated but did not eliminate these apparent disparities, with non-White individuals remaining less likely to be investigated or admitted when found to have a GI disorder.

Ethnicity was generally extremely well-coded, better than has been previously observed in English hospitals [27], and the proportion of attendees from non-White backgrounds was higher than that observed for the population across England overall, in keeping with local population demographics [12]. Minority ethnic attendees tended to be younger than White attendees, most likely explained by the minority ethnic populations in Leicester being generally younger than the White population, although differences in access to healthcare services and cultural attitudes may also contribute. The data also show a significant dip in absolute numbers of patients attending with a GI disorder during and post-COVID which had not fully recovered by the end of 2021. This pattern reflects the overall ED activity levels reported across England post-COVID [28].

Both the initial DPS assigned using a local prioritisation system to determine the required urgency of intervention or secondary assessment and the initial EWS [18, 19] measured in attendees on arrival at the ED were utilised to assess the impact of illness acuity on patient outcomes. By both measures, minority ethnic patients were generally assessed as lower clinical priority than White attendees on arrival at the ED. This observation, and that of minority ethnic patients having shorter time spent in ED, is consistent with a higher proportion of minority ethnic patients being younger and likely attending ED with less serious conditions. However, caution is required in interpreting this non-adjusted data since severity of illness at presentation and likelihood of admission will impact on time spent in ED.

We found lower illness acuity scores in those from ethnic minority groups. However, after adjusting for EWS, rather than DPS because the former score is more widely validated, and other covariables including age, sex and deprivation index, minority ethnic patients remained significantly less likely to be investigated or admitted to hospital than White people for the same types of illness. The reasons are unclear but may relate to cultural differences in illness presentation and healthcare-seeking behaviour between ethnic groups [29, 30]. Disparities in emergency care have previously been described in the US and Australia, where Black or indigenous patients in the ED are less likely to receive clinical imaging investigations and analgesia for acute pain than Whites [23–26]. The bulk of evidence on healthcare outcome inequalities focuses on ethnicity, sex and socioeconomic status [31, 32]; however, when we corrected for both sex and socioeconomic status, racial disparities remained in the current study. Others have attributed such disparities, in part, to implicit bias in the assessment by the healthcare provider [33, 34], although we have no evidence for this and cannot correct for it as a covariable. Underlying reasons are likely to be complex. Differences between ethnic groups in the presentation of illness, the language used to describe symptoms and in illness behaviour are well recognised [29, 30]. Generalised musculoskeletal pain is more common in South Asian individuals in the UK [35]. South Asians with acute coronary syndromes report pain over a larger area of their bodies than White ethnicities [36] and display a greater tendency to seek immediate care [37]. Presentations to ED with GI disorders may also potentially be driven by these factors.

There may also be a mismatch between the clinician’s and the patient’s mental model of disease which impairs communication. Use of language is important in the clinician/patient interaction, but English proficiency is not recorded, so it could not be used as a covariate. Cultural alignment between clinician and patient might also impact the quality of the clinical consultation. Given all of these potential causes of bias, it is reassuring that minority ethnic individuals did not experience a higher rate of ED re-attendance and so were not being discharged prematurely with an illness that subsequently required further assessment or treatment in the future.

The implication might be that White patients were being over-admitted rather than minority ethnic patients being under-admitted. Over-investigation and over-treatment of patients in healthcare is widely recognised, including in emergency medicine [38]. Decisions guiding the investigation and admission of patients may be driven by multiple factors beyond assessment of acuity of presentation, but being subjected to unnecessary investigations or being admitted to hospital unnecessarily would be negative healthcare outcomes, particularly for older people [39, 40]. Lower rates of diagnostic investigations and admissions in the non-White patients may potentially result from these significantly younger individuals having greater engagement in decision making about their care, and with parents able to care for children, coupled with a reluctance on the part of medical staff to discharge the more elderly White patients home from the ED due to a lack of social support.

The strengths of the current analysis include the assembly a large, well-defined cohort, longitudinal over 4 years pre- and post-COVID-19 pandemic, assessed and treated using consistent and standardised processes and the ability to adjust for multiple covariables including illness acuity by two different methods. The wide range of underlying clinical conditions and large differences in age and severity might make severity adjustment incomplete, leaving residual confounding. Data completeness was good and patient ethnicity was reliably collected and very well coded in clinical systems, although aggregated ethnicity categories may not reflect intra-group heterogeneity. That the data are derived from a single centre is a potential limit to generalisability. Data relating to attendees’ presenting symptoms and any co-morbidities were also not available but could have helped interpretation of the findings. Patient disease outcomes (recovery from disease and associated healthcare costs) were not available, so no conclusions could be reached about the effects of the disparities found. Future research will examine disease outcomes for this cohort to enable further understanding of the differences found in this analysis.

We also did not explore interactions between variables such as age and ethnicity as part of this work as this would lack statistical power for detecting differences in outcomes for the smaller groups, thus increasing the risk of type II error. However, future work with a larger sample, or with a more equal distribution across ethnic groups, should explore interactions, especially those between age and/or illness severity with ethnicity.

## Conclusions

The current analysis reveals important new information about differences in the outcomes of acute care for patients from different ethnic groups treated in a single centre. The explanation for these differences is likely to be complex and will require future research to fully elucidate. This will require analysis of whether differences in the processes of care lead to different disease outcomes, by using linked primary and secondary care data and qualitative analyses of attitudes and experiences of both the patients accessing acute care and the staff providing their care.

### Supplementary Information

Additional file 1: Table S1. Record Checklist. Table S2. Diagnostic codes for GI disorders studied. Table 3. Derivation of exposure and outcome variables. Table S4. Logistic regression models showing the relationship between ethnicity and other demographic and clinical parameters with having investigations performed following attendance at the emergency department with GI disorders. Figure S1. Logistic regression models showing the relationship between ethnicity and other demographic and clinical parameters with having investigations performed following attendance at the emergency department with GI disorders. Figure S2. Logistic regression models showing the relationship between ethnicity and other demographic and clinical parameters with admission to hospital following attendance at the emergency department with GI disorders. Figure S3. Complete Case sensitivity analysis—Logistic regression models showing the relationship between ethnicity and other demographic and clinical parameters with having investigations performed following attendance at the emergency department with GI disorders in complete cases. Figure S4. Complete Case sensitivity analysis—Logistic regression models showing the relationship between ethnicity and other demographic and clinical parameters with admission following attendance at the emergency department with GI disorders in complete cases.

## Data Availability

Data will not be shared since this is a service evaluation. Please direct enquiries to the corresponding author.

## Abbreviations

aOR: Adjusted odds ratio
CI: Confidence interval
DPS: Dynamic priority score
ED: Accident and Emergency Department
EWS: Early warning score
GI: Gastrointestinal
IMD: Index of multiple deprivation
IQR: Interquartile range
UHL: University Hospitals of Leicester NHS Trust

## References

[1] The NHS Long Term Plan. 2019. https://www.longtermplan.nhs.uk/wp-content/uploads/2019/08/nhs-long-term-plan-version-1.2.pdf. Accessed 28 Mar 2024.

[2] Hayanga B, Stafford M, Bécares L (2021). Ethnic inequalities in healthcare use and care quality among people with multiple long-term health conditions living in the United Kingdom: a systematic review and narrative synthesis. Int J Environ Res Public Health.

[3] Karlsen S, Nazroo JY (2010). Religious and ethnic differences in health: evidence from the health surveys for England 1999 and 2004. Ethn Health.

[4] Pham TM, Carpenter JR, Morris TP, Sharma M, Petersen I (2019). Ethnic differences in the prevalence of type 2 diabetes diagnoses in the UK: cross-sectional analysis of the health improvement network primary care database. Clin Epidemiol.

[5] Oldroyd J, Banerjee M, Heald A, Cruickshank K (2005). Diabetes and ethnic minorities. Postgrad Med J.

[6] Bansal N, Fischbacher CM, Bhopal RS, Brown H, Steiner MFC, Capewell S (2013). Myocardial infarction incidence and survival by ethnic group: Scottish Health and Ethnicity Linkage retrospective cohort study. BMJ Open.

[7] George J, Mathur R, Shah AD (2017). Ethnicity and the first diagnosis of a wide range of cardiovascular diseases: associations in a linked electronic health record cohort of 1 million patients. PLoS ONE.

[8] Wan YI, Robbins AJ, Apea VJ (2021). Ethnicity and acute hospital admissions: multi-center analysis of routine hospital data. E Clin Med.

[9] Petersen J, Kandt J, Longley PA (2021). Ethnic inequalities in hospital admissions in England: an observational study. BMC Public Health.

[10] Balker C. Accident and emergency statistics: demand, performance and pressure. Briefing Paper, House of Commons Library, 2017. https://researchbriefings.files.parliament.uk/documents/SN06964/SN06964.pdf Accessed 28 Mar 2024.

[11] Office for National Statistics. 2022: https://www.ons.gov.uk/peoplepopulationandcommunity/populationandmigration/populationestimates/datasets/populationandhouseholdestimatesenglandandwalescensus2021 Accessed 28 Mar 2024.

[12] Office for National Statistics. 2022: https://www.ons.gov.uk/visualisations/censusareachanges/E06000016/. Accessed 28 Mar 2024.

[13] Leicestershire County 2021 Census Area Profile. https://www.nomisweb.co.uk/sources/census_2021/report?compare=E10000018. Accessed 3 Apr 2024.

[14] Benchimol EI, Smeeth L, Guttmann A (2015). The REporting of studies Conducted using Observational Routinely-collected health Data (RECORD) Statement. PLoS Med.

[15] Office for National Statistics. Ethnic group, national identity and religion. https://www.ons.gov.uk/methodology/classificationsandstandards/measuringequality/ethnicgroupnationalidentityandreligion. Accessed 28 Mar 2024.

[16] Handbook to the NHS Constitution. Available at: https://www.gov.uk/government/publications/supplements-to-the-nhs-constitution-for-england/the-handbook-to-the-nhs-constitution-for-england. Accessed 3 Apr 2024.

[17] Ministry of Housing, Communities and Local Government. English Indices of Deprivation 2019 UK. https://www.gov.uk/government/statistics/english-indices-of-deprivation-2019. Accessed 28 Mar 2024.

[18] Royal College of Physicians (2017). National Early Warning Score (NEWS) 2: Standardising the assessment of acute-illness severity in the NHS.

[19] Roland D, Stilwell PA, Fortune PM, Alexander J, Clark SJ, Kenny S (2021). Case for change: a standardised inpatient paediatric early warning system in England. Arch Dis Child.

[20] Care Quality Commission. Leicester Royal Infirmary Quality Report 2016. Available at: https://www.leicestershospitals.nhs.uk/EasysiteWeb/getresource.axd?AssetID=47660&type=full&servicetype=Attachment. Accessed 3 Apr 2024.

[21] Rubin DB (1976). Inference and missing data. Biometrika.

[22] Marmot M, Allen J, Boyce T, Goldblatt P, Morrison J. Health Equity in England: The Marmot Review 10 Years On. Institute of Health Equity; 2020. https://health.org.uk/publications/reports/the-marmot-review-10-years-on. 10.1136/bmj.m693 32094110

[23] Shah AA, Zogg CK, Zafar SN, Schneider EB, Cooper LA, Chapital AB (2015). Analgesic access for acute abdominal pain in the emergency department among racial/ethnic minority patients: a nationwide examination. Med Care.

[24] Pletcher MJ, Kertesz SG, Kohn MA, Gonzales R (2008). Trends in opioid prescribing by race/ethnicity for patients seeking care in US emergency departments. JAMA.

[25] Lee P, Maxine Le Saux M, Rebecca Siegel R, Monika Goyal M, Chen C, Ma Y, Meltzer AC (2019). Racial and ethnic disparities in the management of acute pain in US emergency departments: meta-analysis and systematic review. Am J Emergency Med.

[26] Schrager JD, Patzer RE, Kim JJ (2019). Racial and ethnic differences in diagnostic imaging utilization during adult emergency department visits in the United States, 2005 to 2014. J Am Coll Radiol.

[27] Scobie S, Spencer J, Raleigh V. Ethnicity coding in English health service datasets. Nuffield Trust 2021. https://www.nuffieldtrust.org.uk/sites/default/files/2021-06/1622731816_nuffield-trust-ethnicity-coding-web.pdf. Accessed 28 Mar 2024.

[28] Baker C. NHS key statistics: England July 2023. House of Commons Library 2023. https://researchbriefings.files.parliament.uk/documents/CBP-7281/CBP-7281.pdf Accessed 28 Mar 2024.

[29] Palmer B, Macfarlane G, Afzal C, Esmail A, Silman A, Lunt M (2007). Acculturation and the prevalence of pain amongst South Asian minority ethnic groups in the UK. Rheumatology.

[30] Ben-Shlomo Y, Naqvi H, Baker I (2008). Ethnic differences in healthcare-seeking behaviour and management for acute chest pain: secondary analysis of the MINAP dataset 2002–2003. Heart.

[31] James CA, Bourgeois FT, Shannon MW (2005). Association of race/ethnicity with emergency department wait times. Pediatrics.

[32] Turner AJ, Francetic I, Watkinson R, Gillibrand S, Sutton M (2022). Socioeconomic inequality in access to timely and appropriate care in emergency departments. J Health Econ.

[33] Ho J, Burbridge H, Raumati I, Khalil R, Hill D, Jones P (2022). Disposition disparities in an urban tertiary emergency department. Emerg Med Australas.

[34] Soares WE, Knowles KJ, Friedmann PD (2019). A thousand cuts: racial and ethnic disparities in emergency medicine. Med Care.

[35] Allison TR, Symmons DPM, Brammah T (2002). Musculoskeletal pain is more generalised among people from ethnic minorities than among white in Greater Manchester. Ann Rheum Dis.

[36] Dubrey SW, Ghonim S, Teoh M (2014). Acute coronary syndromes among South Asian subgroups in the UK: symptoms and epidemiology Br. J Cardiol.

[37] Chaturvedi N, Rai H, Ben-Shlomo Y (1997). Lay diagnosis and health-care-seeking behaviour for chest pain in south Asians and Europeans. Lancet.

[38] Newton EH (2017). Addressing overuse in emergency medicine: evidence of a role for greater patient engagement. Clin Exp Emerg Med.

[39] San Jose-Saras D, Vicente-Guijarro J, Sousa P (2023). Inappropriate hospital admission as a risk factor for the subsequent development of adverse events: a cross-sectional study. BMC Med.

[40] Schattner A (2023). The spectrum of hospitalization-associated harm in the elderly. Eur J Int Med.

