# Supplementary material for: Ethnicity and outcomes for patients with gastrointestinal disorders attending an emergency department serving a multi-ethnic population
Source: BMC Med. 2024 Jul 2;22:275. doi: 10.1186/s12916-024-03490-0 (PMC11218405; doi:10.1186/s12916-024-03490-0)
Supplement: Supplementary file 1 — Additional file 1: Table S1. Record Checklist. Table S2. Diagnostic codes for GI disorders studied. Table 3. Derivation of exposure and outcome variables. Table S4. Logistic regression models showing the relationship between ethnicity and other demographic and clinical parameters with having investigations performed following attendance at the emergency department with GI disorders. Figure S1. Logistic regression models showing the relationship between ethnicity and other demographic and clinical parameters with having investigations performed following attendance at the emergency department with GI disorders. Figure S2. Logistic regression models showing the relationship between ethnicity and other demographic and clinical parameters with admission to hospital following attendance at the emergency department with GI disorders. Figure S3. Complete Case sensitivity analysis—Logistic regression models showing the relationship between ethnicity and other demographic and clinical parameters with having investigations performed following attendance at the emergency department with GI disorders in complete cases. Figure S4. Complete Case sensitivity analysis—Logistic regression models showing the relationship between ethnicity and other demographic and clinical parameters with admission following attendance at the emergency department with GI disorders in complete cases. [file 12916_2024_3490_MOESM1_ESM.docx]

**ADDITIONAL FILE 1**

**Table S1 Record Checklist**

Ethnicity and Outcomes for Patients with Gastrointestinal Disorders Attending an Emergency Department Serving a Multi-Ethnic Population

The RECORD statement – checklist of items, extended from the STROBE statement, that should be reported in observational studies using routinely collected health data.

|  | **Item No.** | **STROBE items** | **Location in manuscript where items are reported** | **RECORD items** | **Location in manuscript where items are reported** |
| --- | --- | --- | --- | --- | --- |
| **Title and abstract** | | | | | |
|  | 1 | (a) Indicate the study’s design with a commonly used term in the title or the abstract (b) Provide in the abstract an informative and balanced summary of what was done and what was found |  | RECORD 1.1: The type of data used should be specified in the title or abstract. When possible, the name of the databases used should be included.  RECORD 1.2: If applicable, the geographic region and timeframe within which the study took place should be reported in the title or abstract.  RECORD 1.3: If linkage between databases was conducted for the study, this should be clearly stated in the title or abstract. | Abstract: Page 2  Abstract: Page 2  N/A |
| **Introduction** | | | | | |
| Background rationale | 2 | Explain the scientific background and rationale for the investigation being reported |  |  | See Introduction for rationale |
| Objectives | 3 | State specific objectives, including any prespecified hypotheses |  |  | See Introduction for rationale |
| **Methods** | | | | | |
| Study Design | 4 | Present key elements of study design early in the paper |  |  | See Methods, Study design and data source |
| Setting | 5 | Describe the setting, locations, and relevant dates, including periods of recruitment, exposure, follow-up, and data collection |  |  | See Methods, Study design and data source |
| Participants | 6 | *(a) Cohort study* - Give the eligibility criteria, and the sources and methods of selection of participants. Describe methods of follow-up  *Case-control study* - Give the eligibility criteria, and the sources and methods of case ascertainment and control selection. Give the rationale for the choice of cases and controls  *Cross-sectional study* - Give the eligibility criteria, and the sources and methods of selection of participants  *(b) Cohort study* - For matched studies, give matching criteria and number of exposed and unexposed  *Case-control study* - For matched studies, give matching criteria and the number of controls per case |  | RECORD 6.1: The methods of study population selection (such as codes or algorithms used to identify subjects) should be listed in detail. If this is not possible, an explanation should be provided.  RECORD 6.2: Any validation studies of the codes or algorithms used to select the population should be referenced. If validation was conducted for this study and not published elsewhere, detailed methods and results should be provided.  RECORD 6.3: If the study involved linkage of databases, consider use of a flow diagram or other graphical display to demonstrate the data linkage process, including the number of individuals with linked data at each stage. | Methods and Supplementary material  See Methods section.  N/A |
| Variables | 7 | Clearly define all outcomes, exposures, predictors, potential confounders, and effect modifiers. Give diagnostic criteria, if applicable. |  | RECORD 7.1: A complete list of codes and algorithms used to classify exposures, outcomes, confounders, and effect modifiers should be provided. If these cannot be reported, an explanation should be provided. | See supplementary materials for list of codes |
| Data sources/ measurement | 8 | For each variable of interest, give sources of data and details of methods of assessment (measurement).  Describe comparability of assessment methods if there is more than one group |  |  | See Methods, Study design and data source |
| Bias | 9 | Describe any efforts to address potential sources of bias |  |  | See Methods, Study design and statistics. |
| Study size | 10 | Explain how the study size was arrived at |  |  | See Methods, Study design and data source |
| Quantitative variables | 11 | Explain how quantitative variables were handled in the analyses. If applicable, describe which groupings were chosen, and why |  |  | See Methods, Study design and data source  Groupings N/A |
| Statistical methods | 12 | (a) Describe all statistical methods, including those used to control for confounding  (b) Describe any methods used to examine subgroups and interactions  (c) Explain how missing data were addressed  (d) *Cohort study* - If applicable, explain how loss to follow-up was addressed  *Case-control study* - If applicable, explain how matching of cases and controls was addressed  *Cross-sectional study* - If applicable, describe analytical methods taking account of sampling strategy  (e) Describe any sensitivity analyses |  |  | See Methods, Statistics section |
| Data access and cleaning methods |  | .. |  | RECORD 12.1: Authors should describe the extent to which the investigators had access to the database population used to create the study population.  RECORD 12.2: Authors should provide information on the data cleaning methods used in the study. | See Methods: Study population  N/A |
| Linkage |  | .. |  | RECORD 12.3: State whether the study included person-level, institutional-level, or other data linkage across two or more databases. The methods of linkage and methods of linkage quality evaluation should be provided. | See Methods, Study design and data source |
| **Results** | | | | | |
| Participants | 13 | (a) Report the numbers of individuals at each stage of the study (*e.g.*, numbers potentially eligible, examined for eligibility, confirmed eligible, included in the study, completing follow-up, and analysed)  (b) Give reasons for non-participation at each stage.  (c) Consider use of a flow diagram |  | RECORD 13.1: Describe in detail the selection of the persons included in the study (*i.e.,* study population selection) including filtering based on data quality, data availability and linkage. The selection of included persons can be described in the text and/or by means of the study flow diagram. | See Methods, Study population and Figure 1. |
| Descriptive data | 14 | (a) Give characteristics of study participants (*e.g.*, demographic, clinical, social) and information on exposures and potential confounders  (b) Indicate the number of participants with missing data for each variable of interest  (c) *Cohort study* - summarise follow-up time (*e.g.*, average and total amount) |  |  | See Methods, Study population and Figure 1. |
| Outcome data | 15 | *Cohort study* - Report numbers of outcome events or summary measures over time  *Case-control study* - Report numbers in each exposure category, or summary measures of exposure  *Cross-sectional study* - Report numbers of outcome events or summary measures |  |  | See Results text and Table 1. |
| Main results | 16 | (a) Give unadjusted estimates and, if applicable, confounder-adjusted estimates and their precision (e.g., 95% confidence interval). Make clear which confounders were adjusted for and why they were included  (b) Report category boundaries when continuous variables were categorized  (c) If relevant, consider translating estimates of relative risk into absolute risk for a meaningful time period |  |  | See Results section text, Table1 and main data Figures for details. |
| Other analyses | 17 | Report other analyses done—e.g., analyses of subgroups and interactions, and sensitivity analyses |  |  | See Results section for details |
| **Discussion** | | | | | |
| Key results | 18 | Summarise key results with reference to study objectives |  |  | Discussion section first paragraph |
| Limitations | 19 | Discuss limitations of the study, taking into account sources of potential bias or imprecision. Discuss both direction and magnitude of any potential bias |  | RECORD 19.1: Discuss the implications of using data that were not created or collected to answer the specific research question(s). Include discussion of misclassification bias, unmeasured confounding, missing data, and changing eligibility over time, as they pertain to the study being reported. | See Discussion final paragraphs |
| Interpretation | 20 | Give a cautious overall interpretation of results considering objectives, limitations, multiplicity of analyses, results from similar studies, and other relevant evidence |  |  | See Discussion section |
| Generalisability | 21 | Discuss the generalisability (external validity) of the study results |  |  | See Discussion section |
| **Other Information** | | | | | |
| Funding | 22 | Give the source of funding and the role of the funders for the present study and, if applicable, for the original study on which the present article is based |  |  | See Funding section |
| Accessibility of protocol, raw data, and programming code |  | .. |  | RECORD 22.1: Authors should provide information on how to access any supplemental information such as the study protocol, raw data, or programming code. | See Availability of data and materials section |

*Reference: Benchimol EI, Smeeth L, Guttmann A, Harron K, Moher D, Petersen I, Sørensen HT, von Elm E, Langan SM, the RECORD Working Committee. The REporting of studies Conducted using Observational Routinely-collected health Data (RECORD) Statement. *PLoS Medicine* 2015; in press.

*Checklist is protected under Creative Commons Attribution ([CC BY](http://creativecommons.org/licenses/by/4.0/)) license.

**Table S2. Diagnostic codes for GI disorders studied.**

| SNOMED_Code | DI_DESCRIPTION | DI_PRIMARY_CODE |
| --- | --- | --- |
| 80967001 | Dental caries (disorder) | K029 |
| 299709002 | Dental abscess (disorder) | K047 |
| 66383009 | Gingivitis (disorder) | K051 |
| 41888000 | Temporomandibular joint disorder (disorder) | K076 |
| 80483009 | Abscess of salivary gland (disorder) | K113 |
| 80567007 | Sialodocholithiasis (disorder) | K115 |
| 235595009 | Gastroesophageal reflux disease (disorder) | K219 |
| 63305008 | Stricture of oesophagus (disorder) | K222 |
| 23387001 | Perforation of oesophagus (disorder) | K223 |
| 79962008 | Diffuse spasm of oesophagus (disorder) | K224 |
| 13200003 | Peptic ulcer (disorder) | K27 |
| 4556007 | Gastritis (disorder) | K297 |
| 367403001 | Pyloric stenosis (disorder) | K311 |
| 74400008 | Appendicitis (disorder) | K37 |
| 396232000 | Inguinal hernia (disorder) | K409 |
| 50063009 | Femoral hernia | K419 |
| 396347007 | Umbilical hernia (disorder) | K429 |
| 236037000 | Incisional hernia (disorder) | K439 |
| 39839004 | Diaphragmatic hernia | K449 |
| 34000006 | Crohn's disease (disorder) | K509 |
| 64766004 | Ulcerative colitis (disorder) | K519 |
| 91489000 | Acute vascular insufficiency of intestine (disorder) | K559 |
| 49723003 | Intussusception of intestine (disorder) | K561 |
| 9707006 | Intestinal volvulus (disorder) | K562 |
| 81060008 | Intestinal obstruction (disorder) | K566 |
| 307496006 | Diverticulitis (disorder) | K579 |
| 10743008 | Irritable bowel syndrome (disorder) | K589 |
| 14760008 | Constipation (disorder) | K590 |
| 30037006 | Anal fissure (disorder) | K602 |
| 82127005 | Perianal abscess (disorder) | K610 |
| 57773001 | Rectal prolapse (disorder) | K623 |
| 56905009 | Perforation of intestine (disorder) | K631 |
| 48661000 | Peritonitis (disorder) | K659 |
| 59927004 | Hepatic failure (disorder) | K729 |
| 128241005 | Inflammatory disease of liver (disorder) | K759 |
| 328383001 | Chronic liver disease (disorder) | K769 |
| 25924004 | Calculus of gallbladder with cholecystitis (disorder) | K801 |
| 30093007 | Calculus of bile duct (disorder) | K805 |
| 76581006 | Cholecystitis (disorder) | K819 |
| 26918003 | Ascending cholangitis (disorder) | K830 |
| 430887001 | Dysfunction of sphincter of Oddi | K838 |
| 197456007 | Acute pancreatitis (disorder) | K859 |
| 235494005 | Chronic pancreatitis (disorder) | K869 |
| 302918009 | Disorder of stoma (disorder) | K914 |
| 309773000 | Complication of gastrostomy (disorder) | K918 |
| 37372002 | Upper gastrointestinal haemorrhage (disorder) | K922 |
| 87763006 | Lower gastrointestinal haemorrhage (disorder) | K922 |
| 12063002 | Rectal haemorrhage (disorder) | K922 |

**Table S3. Derivation of exposure and outcome variables**

| **Ethnicity (five-level categorical variable)** | Upon contact with hospital services patients are asked to choose the ethnic group with which they identify from a list of 16 ethnic groups. Patients can also choose not to respond which is coded as “not stated”.  Responses were collapsed into the following five categories based on the Office for National Statistics categories:   - **White** - "White - British", "White - Irish", " White - Any other White background" - **Asian** - "Asian - Indian", "Asian - Pakistani", "Asian - Bangladeshi", " Asian - Other", "Chinese" - **Black** - "Black Caribbean", " Black African", "Black Other" - **Mixed** - "Mixed - White & Black Caribbean", "Mixed - White & Black African", "Mixed White & Asian", "Mixed - Other" - **Other** – “Other ethnic group”   Those that responded “Not stated” were coded as missing. |
| --- | --- |
| **Admitted (binary outcome variable)** | Each attendance was flagged with an indicator “Admitted” or “Not admitted” that indicated whether admission to hospital occurred after an attendance at the ED. This variable was used to derive a binary outcome variable (1=admitted vs 0 = not admitted). |
| **Investigated (binary outcome variable)** | A dataset of the number and type of investigation for each visit to ED was extracted from the electronic hospital record. These data were linked to the master dataset using a unique ‘visit ID’ such that for each patient’s first/only attendance at the ED during the study period, a set of investigations relating to this visit were linked to the admission and demographic data. Each unique investigation was then given a numeric code as follows: 1"Amylase", 2"Arterial / capillary blood gas", 3"Bacteriology", 4"Biochemistry", 5"Blood culture", 6"Bone profile", 7"C reactive protein (CRP)", 8"Cardiac enzymes", 9"Clotting studies", 10"Computerised Tomography", 11"Creatine kinase", 12"Cross match blood / group and save serum for later cross match", 13"D-dimer", 14"Dementia screening test", 15"Echocardiography", 16"Electrocardiogram", 17"Glucose", 18"Glycosolated haemoglobin (HbA1c)", 19"Haematology", 20"Image intensifier", 21"Lactate", 22"Lipase", 23"Lipid profile", 24"Liver function tests (LFTs)", 25"Magnetic Resonance Imaging", 26"No investigations performed", 27"Peak expiratory flow", 28"Pregnancy test", 29"Serology", 30"Swab for culture and sensitivities", 31"Thyroid function tests", 32"Toxicology", 33"Troponin", 34"Ultrasound", 35"Urinalysis", 36"Venous blood gas", 37"X-ray plain film", 38"Other", 39"Immunology" 40"mast cell tryptase", 41 "Erythrocyte sedimentation rate", 42"Visual acuity testing", 43"Thromboelastography".  A count of the number of investigations performed was generated. This was then collapsed into a binary variable (1=investigations performed vs 0=no investigations performed). |

**Figure S1.** Logistic regression models showing the relationship between ethnicity and other demographic and clinical parameters with having investigations performed following attendance at the emergency department with GI disorders.


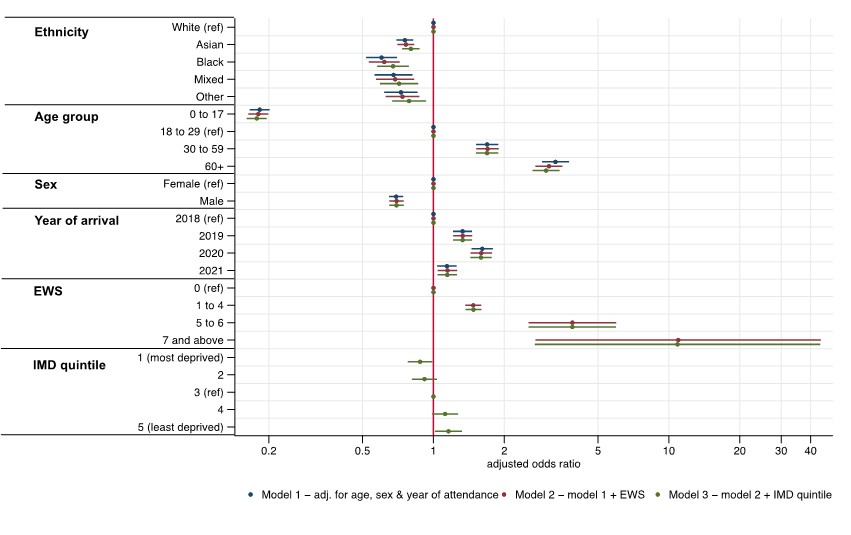


Figure S1 shows the adjusted odds ratios and 95% confidence intervals for the relationship between ethnicity and other covariates with undergoing any investigations after presentation to the emergency department with abdominal pain (n=34,296) using the imputed dataset. Three models were constructed, the first (blue) is adjusted for age, sex and the year of first attendance at the ED with abdominal pain during the study period. The second (red) is additionally adjusted for EWS. The third (green) is additionally adjusted for the IMD quintile. Estimates are represented by dots and the 95% confidence interval for the estimate by bars. x axis is on a log scale.

EWS – Early Warning Score; IMD – Index of Multiple Deprivation; Ref – reference level for categorical/binary covariates

**Figure S2.** Logistic regression models showing the relationship between ethnicity and other demographic and clinical parameters with admission to hospital following attendance at the emergency department with GI disorders.


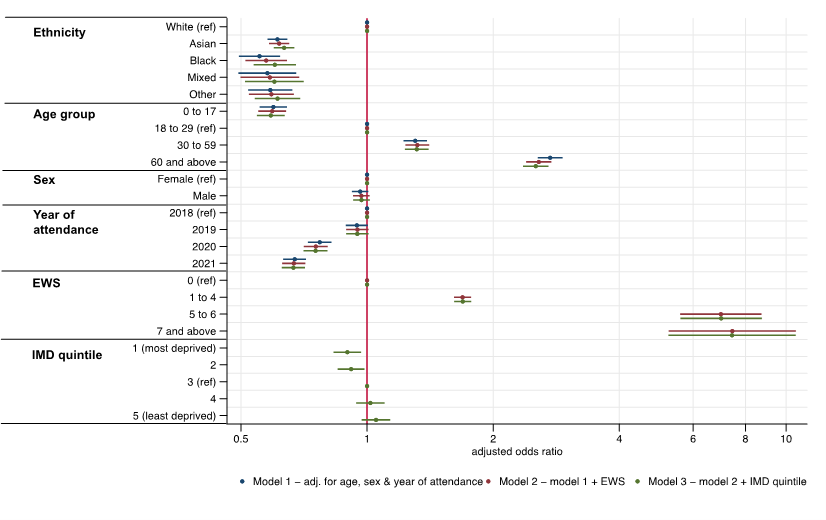


Figure S2 shows the adjusted odds ratios and 95% confidence intervals for the relationship between ethnicity and other covariates with admission to hospital after presentation to the emergency department with Gi disorders (n=34,337) using the imputed dataset. Three models were constructed, the first (blue) is adjusted for age, sex and the year of first attendance at the ED with abdominal pain during the study period. The second (red) is additionally adjusted for EWS. The third (green) is additionally adjusted for the IMD quintile. Estimates are represented by dots and the 95% confidence interval for the estimate by bars. x axis is on a log scale.

EWS – Early Warning Score; IMD – Index of Multiple Deprivation; Ref – reference level for categorical/binary covariates

**Complete Case Analysis**

Figure S3. Sensitivity analysis - Logistic regression models showing the relationship between ethnicity and other demographic and clinical parameters with having investigations performed following attendance at the emergency department with GI disorders in complete cases.


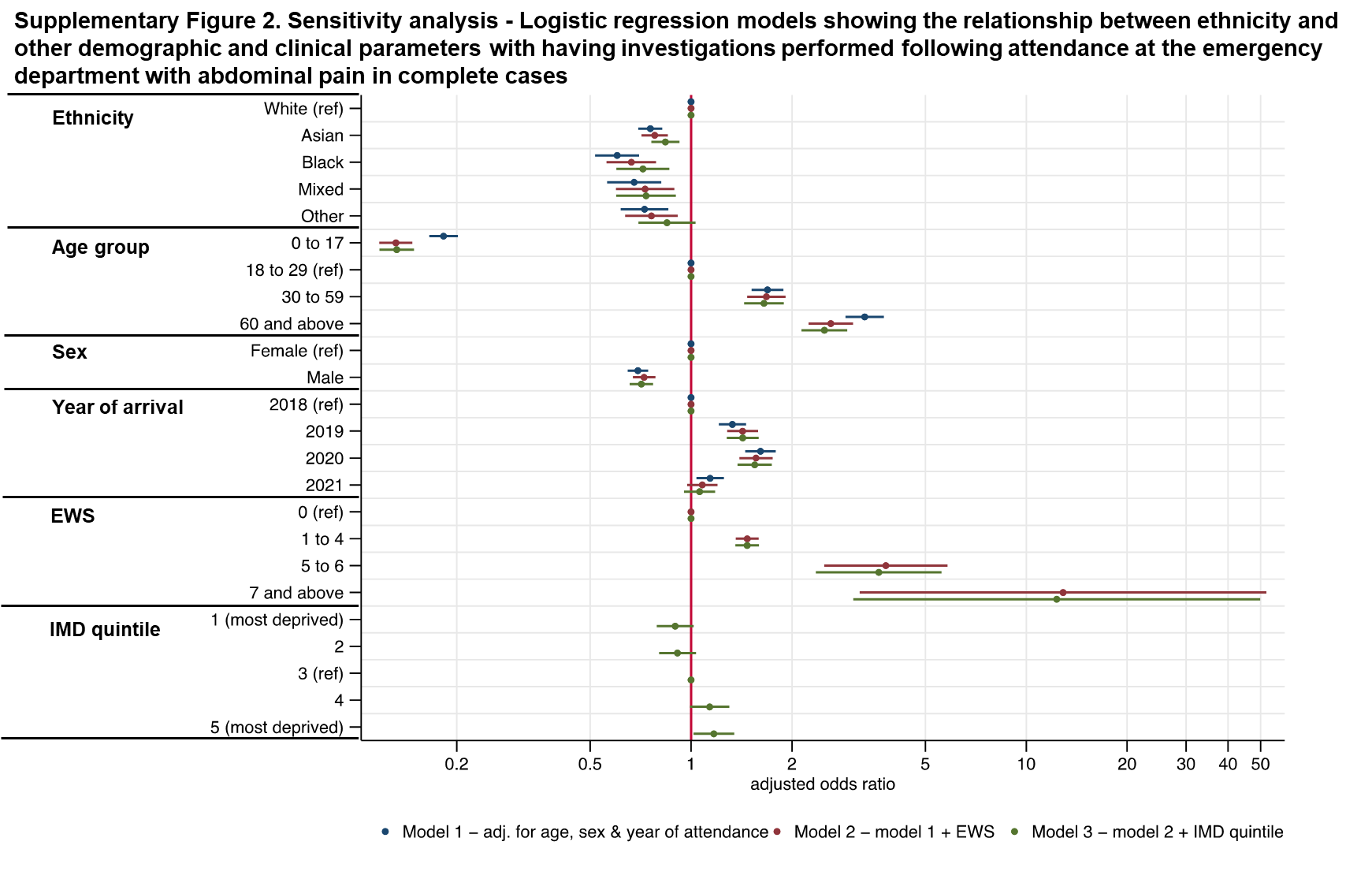


Figure S3 shows the adjusted odds ratios and 95% confidence intervals for the relationship between ethnicity and other covariates with undergoing any investigations after presentation to the emergency department with GI disorders using only complete cases. Three models were constructed, the first (blue, n=34,294) is adjusted for age, sex and the year of first attendance at the ED with abdominal pain during the study period. The second (red, n=30,781) is additionally adjusted for EWS. The third (green, n=29,697) is additionally adjusted for the IMD quintile. Estimates are represented by dots and the 95% confidence interval for the estimate by bars. x axis is on a log scale.

EWS – Early Warning Score; IMD – Index of Multiple Deprivation; Ref – reference level for categorical/binary covariates

Figure S4. Sensitivity analysis - Logistic regression models showing the relationship between ethnicity and other demographic and clinical parameters with admission following attendance at the emergency department with GI disorders in complete cases.


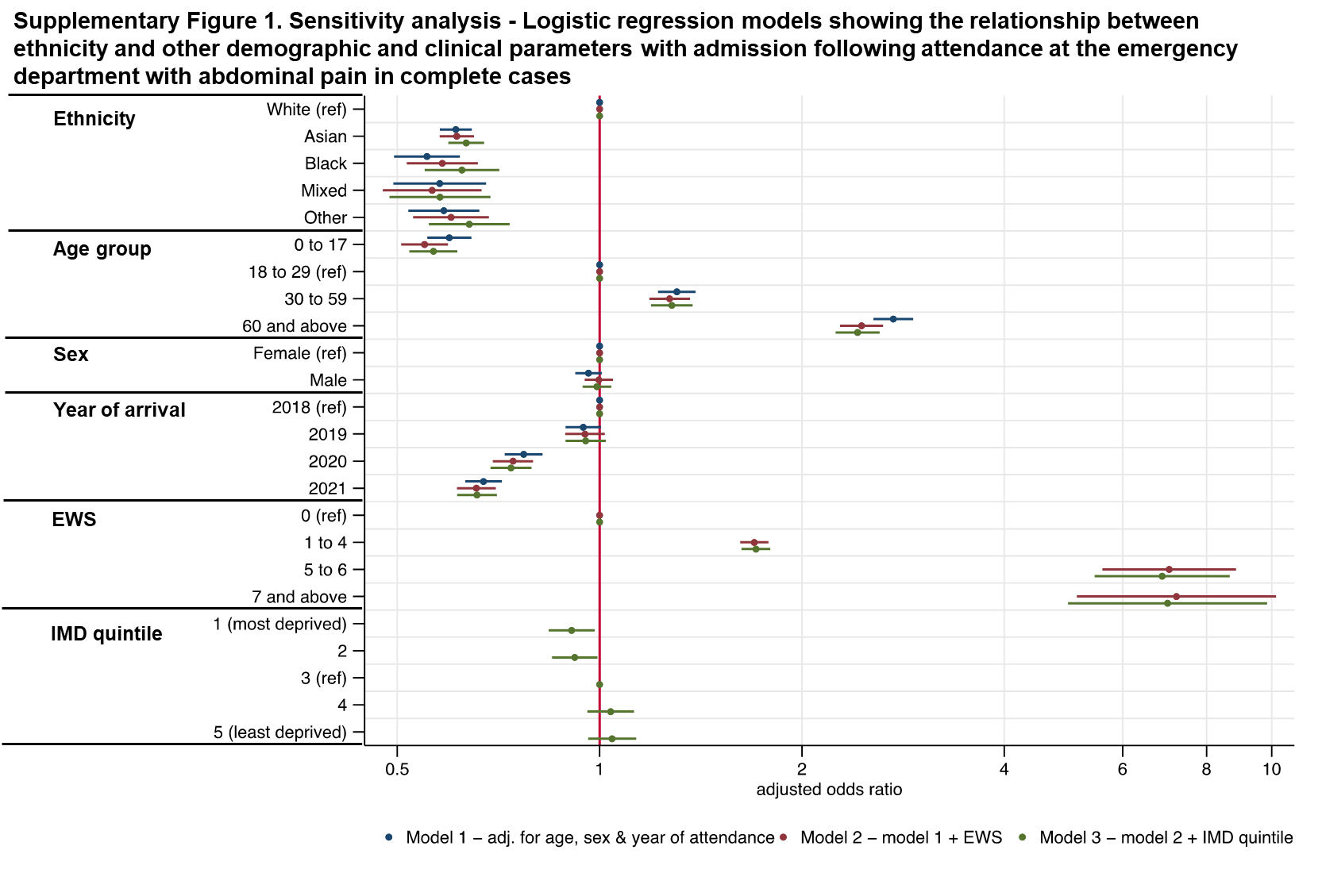


Figure S4 shows the adjusted odds ratios and 95% confidence intervals for the relationship between ethnicity and other covariates with admission to hospital after presentation to the emergency department with GI disorders using only complete cases. Three models were constructed, the first (blue, n=34,335) is adjusted for age, sex and the year of first attendance at the ED with abdominal pain during the study period. The second (red, n=30,812) is additionally adjusted for EWS. The third (green, n=29,728) is additionally adjusted for the IMD quintile. Estimates are represented by dots and the 95% confidence interval for the estimate by bars. x axis is on a log scale.

EWS – Early Warning Score; IMD – Index of Multiple Deprivation; Ref – reference level for categorical/binary covariates

**Table S4. Logistic regression models showing the relationship between ethnicity and other demographic and clinical parameters with having investigations performed following attendance at the emergency department with GI disorders.**

| **Variable** | **Model 1 – adj. for age, sex and year of attendance** | | **Model 2 – model 1 + EWS** | | **Model 3 – model 2 + IMD quintile** | |
| --- | --- | --- | --- | --- | --- | --- |
|  | **Adjusted odds ratio (95% confidence interval)** | **P value** | **Adjusted odds ratio (95% confidence interval)** | **P value** | **Adjusted odds ratio (95% confidence interval)** | **P value** |
| **Ethnicity**  White  Asian  Black  Mixed  Other | Ref  0.76 (0.70 – 0.82)  0.60 (0.52 – 0.70)  0.68 (0.56 – 0.82)  0.73 (0.62 – 0.86) | -  <0.0001  <0.0001  <0.0001  0.0001 | Ref  0.76 (0.70 – 0.83)  0.62 (0.53 – 0.72)  0.69 (0.57 – 0.83)  0.74 (0.63 – 0.87) | -  <0.0001  <0.0001  0.0001  0.0003 | Ref  0.80 (0.74 – 0.87)  0.67 (0.58 – 0.79)  0.71 (0.59 – 0.86)  0.79 (0.67 – 0.93) | -  <0.0001  <0.0001  0.0004  0.0052 |
| **Age group**  0 to 17  18 to 29  30 to 59  60 and above | 0.18 (0.17 – 0.20)  Ref  1.69 (1.52 – 1.89)  3.30 (2.89 – 3.76) | <0.0001  -  <0.0001  <0.0001 | 0.18 (0.16 – 0.20)  Ref  1.70 (1.52 – 1.89)  3.10 (2.71 – 3.53) | <0.0001  -  <0.0001  <0.0001 | 0.18 (0.16 – 0.20)  Ref  1.69 (1.51 – 1.88)  3.01 (2.63 – 3.43) | <0.0001  -  <0.0001  <0.0001 |
| **Sex**  Female  Male | Ref  0.69 (0.65 – 0.74) | -  <0.0001 | Ref  0.70 (0.65 – 0.75) | -  <0.0001 | Ref  0.70 (0.65 – 0.75) | -  <0.0001 |
| **Year of attendance**  2018  2019  2020  2021 | Ref  1.33 (1.21 – 1.46)  1.61 (1.45 – 1.79)  1.14 (1.04 – 1.25) | -  <0.0001  <0.0001  0.007 | Ref  1.33 (1.21 – 1.46)  1.60 (1.44 – 1.77)  1.15 (1.04 – 1.26) | -  <0.0001  <0.0001  0.005 | Ref  1.33 (1.21 – 1.46)  1.59 (1.43 – 1.77)  1.14 (1.04 – 1.26) | -  <0.0001  <0.0001  0.006 |
| **EWS**  0  1 to 4  5 to 6  7 and above |  |  | Ref  1.47 (1.37 – 1.60)  3.89 (2.53 – 5.97)  10.95 (2.71 – 44.19) | -  <0.0001  <0.0001  0.0009 | Ref  1.48 (1.37 – 1.60)  3.89 (2.53 – 5.97)  10.87 (2.69 – 43.89) | -  <0.0001  <0.0001  0.0009 |
| **IMD quintile**  1 (most deprived)  2  3  4  5 (least deprived) |  |  |  |  | 0.88 (0.78 – 0.99)  0.92 (0.81 – 1.04)  Ref  1.12 (0.99 – 1.27)  1.16 (1.01 – 1.32) | 0.03  0.16  -  0.08  0.03 |

**Supplementary Table 3 shows the adjusted odds ratios, 95% confidence intervals and p values for the relationship between ethnicity and other covariates with having investigations performed after presentation to the emergency department with a GI disorder (n=34,337) using the imputed dataset (i.e. the same data presented graphically in Figure 3 and Supplementary Figure 3). Three models were constructed, the first is adjusted for age, sex and the year of first attendance at the ED with abdominal pain during the study period. The second is additionally adjusted for EWS. The third is additionally adjusted for the IMD quintile. EWS – Early Warning Score; IMD – Index of Multiple Deprivation; Ref – reference level for categorical/binary covariates**

**Table S5. Logistic regression models showing the relationship between ethnicity and other demographic and clinical parameters with admission to hospital following attendance at the emergency department with GI disorders.**

| **Variable** | **Model 1 – adj. for age, sex and year of attendance** | | **Model 2 – model 1 + EWS** | | **Model 3 – model 2 + IMD quintile** | |
| --- | --- | --- | --- | --- | --- | --- |
|  | **Adjusted odds ratio (95% confidence interval)** | **P value** | **Adjusted odds ratio (95% confidence interval)** | **P value** | **Adjusted odds ratio (95% confidence interval)** | **P value** |
| **Ethnicity**  White  Asian  Black  Mixed  Other | Ref  0.61 (0.58 – 0.65)  0.55 (0.49 – 0.62)  0.58 (0.49 – 0.68)  0.59 (0.52 – 0.66) | -  <0.0001  <0.0001  <0.0001  <0.0001 | Ref  0.62 (0.58 – 0.65)  0.57 (0.51 – 0.64)  0.59 (0.50 – 0.69)  0.59 (0.52 – 0.67) | -  <0.0001  <0.0001  <0.0001  <0.0001 | Ref  0.63 (0.60 – 0.67)  0.60 (0.54 – 0.68)  0.60 (0.51 – 0.71)  0.61 (0.54 – 0.69) | -  <0.0001  <0.0001  <0.0001  <0.0001 |
| **Age group**  0 to 17  18 to 29  30 to 59  60 and above | 0.60 (0.55 – 0.64)  Ref  1.30 (1.22 – 1.39)  2.73 (2.55 – 2.93) | <0.0001  -  <0.0001  <0.0001 | 0.59 (0.55 – 0.64)  Ref  1.32 (1.24 – 1.41)  2.59 (2.40 – 2.75) | <0.0001  -  <0.0001  <0.0001 | 0.59 (0.55 – 0.64)  Ref  1.31 (1.23 – 1.40)  2.53 (2.36 – 2.71) | <0.0001  -  <0.0001  <0.0001 |
| **Sex**  Female  Male | Ref  0.96 (0.92 – 1.01) | -  0.10 | Ref  0.97 (0.93 – 1.01) | -  0.18 | Ref  0.97 (0.93 – 1.02) | -  0.18 |
| **Year of attendance**  2018  2019  2020  2021 | Ref  0.95 (0.89 – 1.00)  0.77 (0.72 – 0.82)  0.67 (0.63 – 0.72) | -  0.07  <0.0001  <0.0001 | Ref  0.95 (0.89 – 1.01)  0.75 (0.71 – 0.81)  0.67 (0.63 – 0.71) | -  0.09  <0.0001  <0.0001 | Ref  0.95 (0.89 – 1.01)  0.75 (0.71 – 0.80)  0.67 (0.63 – 0.71) | -  0.08  <0.0001  <0.0001 |
| **EWS**  0  1 to 4  5 to 6  7 and above |  |  | Ref  1.69 (1.61 – 1.77)  6.99 (5.59 – 8.74)  7.44 (5.24 – 10.55) | -  <0.0001  <0.0001  <0.0001 | Ref  1.69 (1.61 – 1.77)  7.00 (5.60 – 8.76)  7.43 (5.24 – 10.55) | -  <0.0001  <0.0001  <0.0001 |
| **IMD quintile**  1 (most deprived)  2  3  4  5 (least deprived) |  |  |  |  | 0.90 (0.83 – 0.97)  0.92 (0.85 – 0.97)  Ref  1.02 (0.94 – 1.10)  1.05 (0.97 – 1.14) | 0.005  0.02  -  0.65  0.22 |

**Supplementary Table 4 shows the adjusted odds ratios, 95% confidence intervals and p values for the relationship between ethnicity and other covariates with admission to hospital after presentation to the emergency department with a GI disorder (n=34,337) using the imputed dataset (i.e. the same data presented graphically in Figure 4 and Supplementary Figure 4. Three models were constructed, the first is adjusted for age, sex and the year of first attendance at the ED with abdominal pain during the study period. The second is additionally adjusted for EWS. The third is additionally adjusted for the IMD quintile. EWS – Early Warning Score; IMD – Index of Multiple Deprivation; Ref – reference level for categorical/binary covariates**
